# Anxiety and depressive disorders in the offspring of mothers with perinatal depressive disorders

**DOI:** 10.1007/s00787-025-02803-9

**Published:** 2025-07-25

**Authors:** Biruk Shalmeno Tusa, Rosa Alati, Getinet Ayano, Kim Betts, Berihun Dachew

**Affiliations:** 1 School of Population Health, Curtin University, Kent Street, Bentley, Perth, Western Australia 6102 Australia; 2 Department of Epidemiology and Biostatistics, College of Health and Medical Sciences, Haramaya University, Haramaya, Ethiopia; 3 School of Public Health, The University of Queensland, Brisbane, QLD Australia

**Keywords:** Anxiety disorders, Depressive disorders, Maternal perinatal depressive disorders, Offspring

## Abstract

Maternal perinatal depression has been linked to increased risk of anxiety and depression in offspring. However, many studies are limited by small sample sizes, self-reported data, and insufficient adjustment for confounders, including comorbid maternal anxiety disorders. Using a large linked administrative health dataset from New South Wales, Australia, this study aimed to examine the risk of anxiety and depressive disorders among offspring of mothers with perinatal depressive disorders, with diagnoses identified using the International Classification of Diseases, Tenth Revision, Australian Modification (ICD-10 AM) codes. Associations were assessed using generalized linear model and propensity score matching. Offspring of mothers with perinatal depressive disorders had a 56% higher risk of depressive disorders (RR = 1.56, 95% CI = 1.05–2.29), though this was not observed after matching (RR = 1.42, 95% CI = 0.93–2.18). Maternal perinatal depressive disorders were associated with a higher risk of anxiety disorders (RR = 2.10, 95% CI = 1.78–2.48), which remained significant after propensity score matching (RR = 1.90, 95% CI = 1.59–2.28). Offspring of mothers with comorbid perinatal depressive and anxiety disorders had a 2.36-fold higher risk of depressive disorders (RR = 2.36, 95% CI = 1.21–4.63), a 2.56-fold higher risk of anxiety disorders (RR = 2.56, 95% CI = 1.89–3.48), and a 3.37-fold higher risk of comorbid depressive and anxiety disorders (RR = 3.37, 95% CI = 1.54–7.36). These findings highlight the increased risk of depressive and anxiety disorders in offspring of mothers with perinatal depressive disorders, with stronger associations observed when maternal depression coexists with anxiety. The results underscore the need for early detection and targeted interventions for maternal perinatal depressive and anxiety disorders to reduce adverse mental health outcomes in offspring.

**Supplementary Information:**

The online version contains supplementary material available at 10.1007/s00787-025-02803-9.

## Introduction

Anxiety and depressive disorders are among the most prevalent mental health conditions, affecting individuals across all age groups and both genders [1]. Depressive disorders are characterised by persistent feelings of sadness, emptiness, or irritability, often accompanied by significant disruptions in daily functioning. Anxiety disorders, on the other hand, are marked by excessive fear, heightened anxiety, and associated behavioural disturbances [2]. Globally, over one in five children and adolescents experience anxiety and depression [3, 4]. Moreover, comorbid cases of anxiety and depression are common in this population, further complicating their mental health challenges [5].

The exact causes of anxiety and depression remain unclear but are thought to arise from a complex interplay of multiple factors. A range of studies has highlighted genetic predisposition and adverse environmental conditions as contributors to an increased risk of these disorders [6–10]. For children and adolescents, key risk factors include maternal psychiatric comorbidities, particularly maternal depression, low socioeconomic status, and experiences of childhood maltreatment [11–14]. Maternal antenatal and postnatal depression—affecting approximately 14–15% of women during pregnancy and the first year postpartum—is one of the major contributors to these risks [15, 16]. This period represents a sensitive phase for both fetal brain development and early parent–child interaction, and exposure to maternal depression during this time has been associated with long-term consequences for child emotional and behavioural development [17–23].

Existing epidemiological research highlights that maternal depression during the antenatal and postnatal periods is associated with an increased risk of anxiety and depressive disorders in offspring [24–32]. For example, a longitudinal study in the UK found that antenatal depression was linked to a higher likelihood of depression in children [29]. Similarly, another cohort study reported that children of mothers with postnatal depression were more than three times as likely to develop anxiety or depression than those whose mothers did not experience postnatal depression [24]. However, several critical limitations are evident in existing studies. First, most studies used self-reported screening questionnaires to measure maternal depression [24–26, 28–32] and offspring anxiety and depression [25, 27, 28, 30], raising concerns about the applicability of these findings to clinical settings. Secondly, small sample sizes in many studies limit statistical power and generalisability, increasing the risk of chance findings and restricting the broader applicability of the results [26–28, 30]. Thirdly, many studies have not sufficiently accounted for crucial confounding factors, such as socioeconomic status [24, 28, 29], obstetric complications [24–27, 29–32], adverse birth outcomes [26, 27, 29, 30, 32], and maternal psychiatric and substance use disorders [24, 27, 29]. These factors could significantly influence or alter the observed relationship between maternal perinatal depression and offspring anxiety and depression, potentially introducing bias. Lastly, most studies have not examined the impact of maternal depression on the risk of comorbid depression and anxiety in offspring or the combined effect of maternal depression and anxiety on offspring mental health [24, 26–29, 31, 32]. This gap is significant given the frequent co-occurrence of depression and anxiety in children and adolescents [5] and the high prevalence of these conditions during the antenatal and postnatal periods in mothers [33].

Our study addressed important gaps in existing research by examining the risk of anxiety and depressive disorders in offspring of mothers with depressive disorders, using a large dataset from New South Wales (NSW), Australia. We applied robust diagnostic criteria, specifically the International Classification of Diseases, Tenth Revision, Australian Modification (ICD-10 AM), to identify maternal depression and offspring depression and anxiety while adjusting for key confounders. Additionally, we assessed the impact of maternal perinatal depression on the risk of comorbid depression and anxiety in offspring, as well as the combined effect of maternal depressive and anxiety disorders on offspring mental health. To enhance comparability between exposed and non-exposed offspring, we employed propensity score matching (PSM). These findings have important implications for healthcare and public health policy, supporting efforts to improve maternal mental health and reduce the risk of depression and anxiety in offspring.

## Methods

### Study design, population and data sources

Data included mother-offspring pairs born between January 2003 and December 2005, with follow-up until 2018, using data linked across the NSW Perinatal Data Collection (PDC), the NSW Admitted Patients Data Collection (APDC), and the Mental Health Ambulatory (MH-AMB). The NSW Centre for Health Record Linkage (CHeReL) linked the three data collections using probabilistic linkage methods. A detailed description of the linkage procedures and the quality assurance protocols is available online [34].

### Measurement of outcomes and exposure

Depressive and anxiety disorders in offspring were the primary outcome variables in this study. Information on these diagnoses was obtained from New South Wales (NSW) administrative datasets, including the Admitted Patient Data Collection (APDC) and the Mental Health Ambulatory (MH-AMB) dataset. Diagnoses included both primary and secondary entries, coded according to the ICD-10-AM classification. Depressive disorders were identified using codes F32–F39. Anxiety disorders were identified using codes F40–F43.9 and F93.0–F93.9, which include phobic anxiety disorders (F40), other anxiety disorders (F41), generalised anxiety disorder (F41.1), obsessive–compulsive disorder (F42), reaction to severe stress and adjustment disorders (F43), post-traumatic stress disorder (F43.1), and specific childhood anxiety disorders (F93.0–F93.9).

Maternal perinatal depressive disorders were extracted from the NSW APDC and MH AMB, including all primary and secondary diagnoses. The identification of maternal perinatal depressive disorders was based on ICD-10 AM diagnostic codes F32 through F39. Antenatal depressive disorder was defined as an episode of care occurring between the estimated date of conception and the delivery date, where the conception date was approximated as the delivery date minus the gestational age at delivery. Postnatal depressive disorder was defined as an episode occurring from the delivery date to 52 weeks postpartum.

### Covariates and confounders

A range of predetermined confounders and covariates were included in the analysis. These included socio-economic indicators, the baby’s sex, birth order, parity, mode of delivery, preterm birth (a live birth occurring before 37 weeks of gestation), low birth weight (birth weight under 2,500 g), low Apgar scores (Apgar scores below seven at five minutes), antenatal anaemia, antenatal maternal infection, pregnancy-induced hypertension, gestational diabetes, preconception depressive disorder (ICD-10 codes F32-F39), antenatal and postnatal anxiety disorder (ICD10 codes F40-F47.9), perinatal bipolar disorder (ICD-10 codes F30-F31.9), perinatal schizophrenia (ICD-10 codes F20-F29), perinatal alcohol use disorder (ICD-10 codes F10-F10.9), and perinatal substance use disorder (ICD-10 codes F11-F17.9). Data for these variables were drawn from the PDC, APDC, and MH AMB datasets.

### Statistical analysis

Pearson chi-square tests were used to compare offspring with and without depressive and anxiety disorders across key socio-economic, maternal, and child-related characteristics. To assess the association between maternal perinatal depressive disorders and the risk of depressive and anxiety disorders in offspring, including both any anxiety disorder and specific types, we employed generalised linear models (GLMs) with a binomial distribution and a log link function. Risk ratios (RRs) with 95% confidence intervals (CIs) were calculated to quantify the strength and statistical significance of the associations. Given the notable comorbidity between depressive and anxiety disorders in children and adolescents, we further examined how maternal perinatal depression was associated with the risk of comorbid depressive and anxiety disorders in offspring [5]. We also assessed the combined impact of maternal depressive and anxiety disorders on the risk of depressive and anxiety disorders in offspring.

We also applied PSM using nearest neighbour matching (NNM) with a 4:1 matching ratio and a caliper of 0.25 to improve comparability between exposed and non-exposed offspring. The propensity score was estimated using a GLM with a binomial distribution, incorporating a range of predetermined covariates and confounders. Matching was conducted without replacement using the MatchIt package in R [35]. To assess balance before and after matching, we examined standardised mean differences (SMDs) in numerical summaries alongside graphical assessments, including Love plots and propensity score distribution plots. Successful balance was indicated by a post-matching SMD below 0.1, covariate differences close to zero on the Love plot, and overlapping distributions with closely aligned peaks in the propensity score distribution plots [35] (Figure S1 to S8). After obtaining the matched data, we fitted GLMs with a binomial distribution and a log link function to assess the association between exposures and outcomes.

## Results

### Characteristics of the study population

The analysis included 223,068 mother-offspring pairs. Of the offspring, 51.6% were male, 98.5% were first in birth order, 6.5% were born preterm, 5.3% had low birth weight, and 1.1% had a low five-minute Apgar score. Overall, 4,552 (2.0%) offspring were diagnosed with an anxiety disorder, 1,098 (0.5%) with a depressive disorder, and 593 (0.3%) with both conditions. Among those with anxiety disorders, 1,704 (0.8%) had reaction to severe stress and adjustment disorders (RSSAD), 543 (0.2%) had generalised anxiety disorder (GAD), 453 (0.2%) had post-traumatic stress disorder (PTSD), and 872 (0.4%) were classified under other anxiety disorders (OAD). Among the mothers, 1,940 (0.9%) were diagnosed with antenatal depressive disorder, 996 (0.5%) with postnatal depressive disorder, and 2,793 (1.3%) with perinatal depressive disorder. Additionally, 559 (0.3%) had comorbid perinatal depressive and anxiety disorders.

Table 1 presents the characteristics of study participants based on offspring depressive and anxiety disorders. Mothers of affected offspring were more likely to have experienced preconception, antenatal, and postnatal depressive disorders; antenatal and postnatal anxiety disorders; perinatal bipolar disorder; schizophrenia; alcohol and substance use disorders; antenatal infections; pregnancy-induced hypertension; and gestational diabetes compared to mothers of unaffected offspring. Additionally, depressive and anxiety disorders were more common in boys than girls, and affected offspring were more likely to have low birth weight.

**Table 1:** Maternal and offspring characteristics by outcome status (*n* = 223,068)

Variable	Offspring Depressive Disorder (			Offspring Anxiety Disorder			Total (%)
	Yes (%) (*n* = 1,098)	No (%) (*n* = 221,970)	*P*-value #	Yes (%) (*n* = 4,552)	No (%) (*n* = 218,516)	*P*-value #	
**Maternal characteristics**							
Maternal age							
12–19	135 (12.3)	12,973 (5.9)	< 0.01	480 (10.6)	12,628 (5.8)	< 0.01	13,108 (5.9)
20–24	170 (15.5)	26,940 (12.1)		696 (15.3)	26,414 (12.1)		27,109 (12.2)
25–29	285 (26.0)	61,172 (27.6)		1,169 (25.7)	60,287 (27.6)		61,457 (27.5)
30–34	291 (26.5)	75,845 (34.1)		1,314 (28.8)	74,822 (34.2)		76,136 (34.1)
>=35	217 (19.7)	45,020 (20.3)		892 (19.6)	44,345 (20.3)		45,237 (20.3)
Socio-Economic Indexes for Area							
1st most-disadvantage	332 (30.2)	55,665 (25.1)	< 0.01	1,235 (27.1)	54,762 (25.1)	< 0.01	55,997 (25.1)
2nd most-disadvantage	281 (25.6)	48,579 (21.9)		1,053 (23.2)	47,807 (21.9)		48,860 (21.9)
3^rd−^ lowest disadvantage	244 (22.2)	54,390 (24.5)		1,048 (23.0)	53,586 (24.5)		54,634 (24.5)
4th lowest disadvantage	241 (22.0)	63,148 (28.5)		1,215 (26.7)	62,174 (28.5)		63,389 (28.5)
Parity							
Nulliparity	445 (40.5)	91,869 (41.4)	0.01	2,007 (44.1)	90,307 (41.3)	< 0.01	92,314 (41.4)
Low multiparity (1–3)	597 (54.4)	122,477 (55.2)		2,350 (51.6)	120,724 (55.3)		123,074 (55.2)
Grand multipara (> 3)	56 (5.1)	7,624 (3.4)		195 (4.3)	7,485 (3.4)		7,680 (3.4)
Mode of delivery							
Normal vaginal	696 (63.4)	136,797 (61.6)	0.06	2,826 (62.1)	134,667 (61.6)	0.24	137,493 (61.7)
Vacuum extraction	66 (6.0)	15,590 (7.0)		288 (6.3)	15,368 (7.0)		15,656 (7.0)
Caesarean section	284 (25.9)	61,597 (27.8)		1,262 (27.7)	60,619 (27.7)		61,881 (27.7)
Others*	52 (4.7)	7,986 (3.6)		176 (3.9)	7,862 (3.6)		8,038 (3.6)
Antenatal maternal infection							
Yes	21 (1.9)	1,715 (0.8)	< 0.01	52 (1.1)	1,684 (0.8)	0.01	1,736 (0.8)
No	1,077 (98.1)	220,255 (99.2)		4,500 (98.9)	216,832 (99.2)		221,332 (99.2)
Pregnancy-induced hypertension							
Yes	97 (8.8)	18,058 (8.1)	0.40	421 (9.3)	17,734 (8.1)	0.01	18,155 (8.1)
No	1,001 (91.2)	203,912 (91.9)		4,131 (90.7)	200,782 (91.9)		204,913 (91.9)
Gestational diabetes							
Yes	63 (5.7)	10,944 (4.9)	0.22	203 (4.5)	10,804 (4.9)	0.14	11,007 (4.9)
No	1,035 (94.3)	211,026 (95.1)		4,349 (95.5)	207,712 (95.1)		212,061 (95.1)
Antenatal anaemia							
Yes	24 (2.2)	4,470 (2.0)	0.69	130 (2.9)	4,364 (2.0)	< 0.01	4,494 (2.0)
No	1,074 (97.8)	217,154 (98.0)		4,422 (97.1)	214,152 (98.0)		218,574 (98.0)
Preconception depressive disorder							
Yes	11 (1.0)	853 (0.4))	< 0.01	46 (1.0)	818(0.4)	< 0.01	864 (0.4)
No	1,087 (99.0)	221,217 (99.6)		4,506 (99.0)	217,698 (99.6)		222,204 (99.6)
Antenatal depressive disorder							
Yes	22 (2.0)	1,918 (0.9)	< 0.01	113 (2.5)	1,827(0.8)	< 0.01	1,940 (0.9)
No	1,076 (98.0)	220,052 (99.1)		4,439 (97.5)	216,689 (99.2)		221,128 (99.1)
Postnatal depressive disorder							
Yes	12 (1.1)	984 (0.4)	< 0.01	70 (1.5)	926 (0.4)	< 0.01	996 (0.5)
No	1,086 (98.9)	220,986 (99.6)		4,482 (98.5)	217,590 (99.6)		222,072 (99.5)
Perinatal depressive disorder							
Yes	30 (2.7)	2,763 (1.2)	< 0.01	170 (3.7)	2,623 (1.2)	< 0.01	2,793 (1.3)
No	1,068 (97.3)	219,207 (98.8)		4,382 (96.3)	215,893 (98.8)		220,275 (98.7)
Antenatal anxiety disorder							
Yes	14 (1.3)	1,374 (0.6)	0.01	75 (1.7)	1,313 (0.6)	< 0.01	1,388 (0.6)
No	1,084 (98.7)	220,596 (99.4)		4,477 (98.4)	217,203 (99.4)		221,680 (99.4)
Postnatal anxiety disorder							
Yes	18 (1.6)	2,233 (1.0)	0.04	107 (2.4)	2,144 (1.0)	< 0.01	2,251 (1.0)
No	1,080 (98.4)	219,737 (99.9)		4,445 (97.6)	216,372 (99.0)		221,625 (99.0)
Perinatal bipolar disorder							
Yes	4 (0.4)	305 (0.1)	0.04	20 (0.4)	289 (0.1)	< 0.01	309 (0.1)
No	1,094 (99.6)	221,665 (99.9)		4,532 (99.6)	218,227 (99.9)		222,759 (99.9)
Perinatal schizophrenia disorder							
Yes	5 (0.5)	352 (0.2)	0.01	21 (0.5)	334 (0.1)	< 0.01	357 (0.2)
No	1,093 (99.5)	221,618 (99.8)		4,529 (99.5)	218,182 (99.9)		222,711 (99.8)
Perinatal alcohol use disorder							
Yes	9 (0.8)	455 (0.2)	< 0.01	25 (0.6)	439 (0.2)	< 0.01	464 (0.2)
No	1,089 (99.2)	221,515 (99.8)		4,527 (99.4)	218,077 (99.8)		222,604 (99.8)
Perinatal substance use disorder*							
Yes	34 (3.1)	2,644 (1.2)	< 0.01	146 (3.2)	2,532 (1.2)	< 0.01	2,678 (1.2)
No	1,064 (96.9)	219,326 (98.8)		4,406 (96.8)	215,984 (98.8)		220,390 (98.8)
**Offspring characteristics**							
Sex of baby							
Male	365 (33.3)	114,678 (51.7)	< 0.01	1,984 (43.6)	113,059 (51.7)	< 0.01	115,043 (51.6)
Female	732 (66.7)	107,151 (48.3)		2,563 (56.4)	105,320 (48.3)		107,883 (48.4)
Birth order							
First	1,081 (98.5)	218,702 (98.5)	0.84	4,483 (98.5)	215,300 (98.5)	0.81	219,783 (98.5)
Second and above	17 (1.5)	3,268 (1.5)		69 (1.5)	3,216 (1.5)		3,285 (1.5)
Preterm							
Yes	68 (6.2)	13,504 (6.1)	0.88	330 (7.3)	13,241 (6.1)	< 0.01	13,571 (6.5)
No	1,030 (93.8)	208,466 (93.9)		4,222 (92.7)	205,275 (93.9)		209,497 (93.5)
Low birth weight							
Yes	75 (6.8)	11,659 (5.3)	0.02	308 (6.8)	11,426 (5.2)	< 0.01	11,734 (5.3)
No	1,022 (93.2)	210,266 (94.8)		4,243 (93.2)	207,045 (94.8)		211,288 (94.7)
Low APGAR score							
Yes	15 (1.4)	2,524 (1.1)	0.48	89 (2.0)	2,450 (1.1)	< 0.01	2,539 (1.1)
No	1,083 (98.6)	219,257 (98.9)		4,463 (98.0)	215,877 (98.9)		220,340 (98.9)

* Others include forceps, vaginal breech, and not stated

**Substance use disorders include tobacco, cannabis, opioids, cocaine, hallucinogens, and stimulants

# Calculated using the Pearson χ2 test

### The risk of depressive disorder in the offspring of mothers with perinatal depressive disorders

After adjusting for confounding factors, offspring of mothers with perinatal depressive disorders had a 56% higher likelihood of being diagnosed with a depressive disorder (RR = 1.56, 95% CI = 1.05–2.29). Specifically, maternal antenatal depressive disorder was associated with a 62% increased likelihood (RR = 1.62, 95% CI = 1.04–2.52), whereas maternal postnatal depressive disorders were not associated with an increased risk (RR = 1.44, 95% CI = 0.78–2.65). In adjusted PSM analyses, maternal antenatal (RR = 1.55, 95% CI = 0.94–2.57), postnatal (RR = 1.56, 95% CI = 0.80–3.05), and overall perinatal depressive disorders (RR = 1.42, 95% CI = 0.93–2.18) were not significantly associated with offspring depressive disorders (Table 2).

**Table 2:** Univariable and multivariable log-binomial analysis for risk of depressive and anxiety disorder in offspring of mothers with depressive disorders

Variables	Offspring depressive disorders				Offspring anxiety disorders			
	Unadjusted RR (95% CI)	*P*-value	Adjusted RR^#^ (95% CI)	*P*-value	Unadjusted RR (95% CI)	*P*-value	Adjusted RR^#^ (95% CI)	*P*-value
Antenatal depressive disorders*								
Unmatched (*N* = 222,628)	2.33 (1.53, 3.55)	**< 0.01**	1.62 (1.04, 2.51)	**0.03**	2.90 (2.42, 3.48)	**< 0.01**	1.87 (1.54, 2.28)	**< 0.01**
Matched (*N* = 9,399)	1.58 (0.95, 2.61)	0.08	1.55 (0.94, 2.57)	0.09	1.92 (1.54, 2.40)	**< 0.01**	1.85 (1.48, 2.31)	**< 0.01**
Postnatal depressive disorders **								
Unmatched (*N* = 222,628)	2.47 (1.40, 4.34)	**< 0.01**	1.44 (0.78, 2.65)	0.24	3.48 (2.77, 4.37)	**< 0.01**	1.82 (1.41, 2.34)	**< 0.01**
Matched (*N* = 4,662)	1.99 (0.98, 4.03)	0.06	1.56 (0.80, 3.05)	0.19	1.89 (1.43, 2.50)	**< 0.01**	1.80 (1.12, 2.19)	**< 0.01**
Perinatal depressive disorder ***								
Unmatched (*N* = 222,628)	2.22 (1.55, 3.18)	**< 0.01**	1.56 (1.05–2.29)	**0.03**	3.06 (2.64, 3.55)	**< 0.01**	2.10 (1.78, 2.48)	**< 0.01**
Matched (*N* = 13,366)	1.45 (0.95, 2.21)	0.09	1.42 (0.93, 2.18)	0.10	1.99 (1.66, 2.39)	**< 0.01**	1.90 (1.59, 2.28)	**< 0.01**
**Comorbid perinatal depressive and** **anxiety disorders**								
Unmatched (*N* = 222,628)	3.32 (1.73, 6.34)	**< 0.01**	2.36 (1.21, 4.63)	**0.01**	3.74 (2.79, 5.00)	**< 0.01**	2.56 (1.89, 3.48)	**< 0.01**
Matched (*N* = 2,688)	2.95 (1.25, 6.98)	< 0.01	3.00 (1.26, 7.14)	**0.01**	2.61 (1.78, 3.82)	**< 0.01**	2.56 (1.75, 3.73)	**< 0.01**

^#^Models adjusted for maternal age, socio-economic indicators, sex of the baby, birth order, parity, preterm, low birth weight, low APGAR score, mode of delivery, antenatal maternal infection, antenatal maternal anaemia, pregnancy-induced hypertension, gestational diabetes, preconception depressive disorder, perinatal bipolar disorder, perinatal schizophrenia disorder, perinatal alcohol use disorder, and perinatal substance use disorder

^*^Further adjusted for postnatal depressive disorder, antenatal anxiety disorder, and postnatal anxiety disorder

^**^Further adjusted for antenatal depressive disorder, antenatal anxiety disorder, and postnatal anxiety disorder

^***^Further adjusted for antenatal anxiety disorder, and postnatal anxiety disorder

### The risk of anxiety disorder in the offspring of mothers with perinatal depressive disorders

In multivariable unmatched analyses, maternal antenatal, postnatal, and overall perinatal depressive disorders were associated with an increased risk of anxiety disorders in offspring, with risk estimates of RR = 1.87 (95% CI = 1.54–2.28), RR = 1.82 (95% CI = 1.41–2.34), and RR = 2.10 (95% CI = 1.78–2.48), respectively. Similar associations were observed in adjusted PSM analyses (RR = 1.85, 95% CI = 1.48–2.31; RR = 1.80, 95% CI = 1.12–2.19; RR = 1.90, 95% CI = 1.59–2.28) (Table 2).

Maternal perinatal depressive disorders were also associated with specific anxiety disorder types in both multivariable unmatched and matched analyses, with an increased risk of offspring GAD (RR = 3.34, 95% CI = 2.20–5.07; RR = 4.13, 95% CI = 1.81–9.42), RSSAD (RR = 2.18, 95% CI = 1.69–2.80; RR = 1.87, 95% CI = 1.22–2.87), PTSD (RR = 2.60, 95% CI = 1.69–4.00; RR = 3.05, 95% CI = 1.32–7.05), and OAD (RR = 1.84, 95% CI = 1.21–2.82; RR = 2.15, 95% CI = 1.02–4.51) (Table 3).

**Table 3:** Univariable and multivariable log-binomial analysis for risk of specific types of anxiety disorders in offspring of mothers with depressive disorders

Outcome variables	Antenatal depressive disorder* Unmated (*N* = 222,628) and Matched (*N* = 9,399)				Postnatal depressive disorder ** Unmated (*N* = 222,628) and Matched (*N* = 4,662)				Perinatal depressive disorder *** Unmated (*N* = 222,628), Matched (*N* = 13,366)			
	Unadjusted RR (95% CI)	*P*-value	Adjusted RR^#^ (95% CI)	*P*-value	Unadjusted RR (95% CI)	*P*-value	Adjusted RR^#^ (95% CI)	*P*-value	Unadjusted RR (95% CI)	*P*-value	Adjusted RR^#^ (95% CI)	*P*-value
**Generalised anxiety disorders**												
Unmatched	5.50 (3.69, 8.20)	**< 0.01**	4.17 (2.69, 6.46)	**< 0.01**	2.49 (1.12, 5.56)	**0.03**	1.02 (0.42, 2.44)	0.97	4.45 (3.07, 6.46)	**< 0.01**	3.34 (2.20, 5.07)	**< 0.01**
Matched	5.52 (2.97, 10.25)	**< 0.01**	5.39 (2.91, 10.00	**< 0.01**	0.90 (0.34, 2.38)	0.83	0.95 (0.36, 2.54)		4.00 (1.79, 8.96)	**< 0.01**	4.13 (1.81, 9.42)	**< 0.01**
**Reaction to severe stress, and adjustment disorders**												
Unmatched	3.38 (2.55, 4.47)	**< 0.01**	1.78 (1.32, 2.42)	**< 0.01**	4.40 (3.14, 6.18)	**< 0.01**	2.05 (1.40, 2.99)	**< 0.01**	3.69 (2.94, 4.63)	**< 0.01**	2.18 (1.69, 2.80)	**< 0.01**
Matched	1.77 (1.26, 2.48)	**< 0.01**	1.71 (1.22, 2.39)	**< 0.01**	2.40 (1.56, 3.69)	**< 0.01**	2.22 (1.44, 3.42)	**< 0.01**	2.07 (1.36, 3.15)	**< 0.01**	1.87 (1.22, 2.87)	**< 0.01**
**Post-traumatic stress disorders**												
Unmatched	5.27 (3.37, 8.22)	**< 0.01**	2.38 (1.46–3.90)	**< 0.01**	6.07 (3.43, 10.73)	**0.02**	2.18 (1.14, 4.17)	**< 0.01**	5.20 (3.55, 7.61)	**< 0.01**	2.60 (1.69, 4.00)	**< 0.01**
Matched	2.40 (1.36, 4.23)	**< 0.01**	2.32 (1.31, 4.10)	**< 0.01**	3.49 (1.60, 7.62)	**< 0.01**	3.30 (1.49, 7.34)	0.01	3.77 (1.67, 8.50)	**< 0.01**	3.05 (1.32, 7.05)	**< 0.01**
**Other anxiety disorders**												
Unmatched	2.27 (1.41, 3.66)	**< 0.01**	1.71 (1.03, 2.83)	**0.04**	2.59 (1.39, 4.81)	**< 0.01**	1.76 (0.90, 3.44)	0.10	2.33 (1.57, 3.46)	**< 0.01**	1.84 (1.21, 2.82)	**0.01**
Matched	1.83 (1.00, 3.38)	**0.05**	1.84 (1.00, 3.40)	**0.05**	1.70 (0.78, 3.72)	0.19	1.57 (0.71, 3.46)	0.27	2.27 (1.10, 4.69)	**0.03**	2.15 (1.02, 4.51)	**0.04**

^#^Models adjusted for maternal age, socio-economic indicators, sex of the baby, birth order, parity, preterm, low birth weight, low APGAR score, mode of delivery, antenatal maternal infection, antenatal maternal anaemia, pregnancy-induced hypertension, gestational diabetes, preconception depressive disorder, perinatal bipolar disorder, perinatal schizophrenia disorder, perinatal alcohol use disorder, and perinatal substance use disorder

^*^Further adjusted for postnatal depressive disorder, antenatal anxiety disorder, and postnatal anxiety disorder

^**^Further adjusted for antenatal depressive disorder, antenatal anxiety disorder, and postnatal anxiety disorder

***Further adjusted for antenatal anxiety disorder, and postnatal anxiety disorder

Offspring of mothers with antenatal depressive disorders had a higher likelihood of experiencing GAD (RR = 4.17, 95% CI = 2.69–6.46), RSSAD (RR = 1.78, 95% CI = 1.32–2.42), PTSD (RR = 2.38, 95% CI = 1.46–3.96), and OAD (RR = 1.71, 95% CI = 1.03–2.83). These associations remained significant in adjusted PSM analyses (Table 3).

In both unmatched and matched analyses, maternal postnatal depressive disorders were significantly associated with an increased risk of offspring developing RSSAD (RR = 2.05, 95% CI = 1.40–2.99; RR = 2.22, 95% CI = 1.44–3.42) and PTSD (RR = 2.18, 95% CI = 1.14–4.17; RR = 3.30, 95% CI = 1.49–7.34). No significant association was observed with GAD (RR = 1.02, 95% CI = 0.42–2.44; RR = 0.95, 95% CI = 0.36–2.54) or OAD (RR = 1.76, 95% CI = 0.90–3.44; RR = 1.57, 95% CI = 0.71–3.46) (Table 3).

### The risk of depressive and anxiety disorders in the offspring of mothers with comorbid perinatal depressive and anxiety disorders

Offspring of mothers with comorbid perinatal depressive and anxiety disorders had more than a two-fold higher risk of depressive disorders (RR = 2.36, 95% CI = 1.21–4.63) and anxiety disorders (RR = 2.56, 95% CI = 1.89–3.48), and more than a three-fold higher risk of comorbid depressive and anxiety disorders (RR = 3.37, 95% CI = 1.54–7.36). These associations remained elevated in PSM analyses (Table 2and Table 4).

**Table 4:** Univariable and multivariable log-binomial analysis for risk of comorbid depressive and anxiety disorder in offspring of mothers with depressive disorders

Variables	Offspring comorbid depressive and anxiety disorders			
	Unadjusted RR (95% CI)	*P*-value	Adjusted RR^#^ (95% CI)	*P*-value
Antenatal depressive disorders*				
Unmatched (*N* = 222,628)	2.96 (1.78, 4.93)	**< 0.01**	2.08 (1.21, 3.57)	**0.01**
Matched (*N* = 9,399)	1.82 (0.97, 3.43)	0.06	1.76 (0.94, 3.30)	0.08
Postnatal depressive disorders **				
Unmatched (*N* = 222,628)	3.05 (1.52, 6.11)	**< 0.01**	1.63 (0.75, 3.52)	0.22
Matched (*N* = 4,662)	1.51 (0.67, 3.42)	0.32	1.36 (0.88, 4.78)	0.47
Perinatal depressive disorder ***				
Unmatched (*N* = 222,628)	2.75 (1.77, 4.30)	**< 0.01**	1.94 (1.20, 3.16)	**0.01**
Matched (*N* = 13,366)	1.87 (1.08, 3.24)	**0.02**	1.78 (1.02, 3.32)	**0.04**
**Comorbid perinatal depressive and** **anxiety disorders**				
Unmatched (*N* = 222,628)	4.79 (2.28, 10.04)	**< 0.01**	3.37 (1.54, 7.36)	**< 0.01**
Matched (*N* = 2,688)	4.60 (1.55, 13.63)	**0.01**	5.44 (1.74, 17.00)	**< 0.01**

^#^Models adjusted for maternal age, socio-economic indicators, sex of the baby, birth order, parity, preterm, low birth weight, low APGAR score, mode of delivery, antenatal maternal infection, antenatal maternal anaemia, pregnancy-induced hypertension, gestational diabetes, preconception depressive disorder, perinatal bipolar disorder, perinatal schizophrenia disorder, perinatal alcohol use disorder, and perinatal substance use disorder

^*^Further adjusted for postnatal depressive disorder, antenatal anxiety disorder, and postnatal anxiety disorder

^**^Further adjusted for antenatal depressive disorder, antenatal anxiety disorder, and postnatal anxiety disorder

***Further adjusted for antenatal anxiety disorder, and postnatal anxiety disorder

### The risk of comorbid depressive and anxiety disorder in the offspring of mothers with perinatal depressive disorders

Maternal perinatal depressive disorders were associated with a 94% increased risk of comorbid depressive and anxiety disorders in offspring (RR = 1.94, 95% CI = 1.20–3.16), which remained significant in adjusted PSM analysis (RR = 1.78, 95% CI = 1.02–3.32). Antenatal depressive disorders were linked to more than twice the risk (RR = 2.08, 95% CI = 1.21–3.57), though this association attenuated after covariate matching (RR = 1.76, 95% CI = 0.94–3.30). No significant association was observed for postnatal depressive disorders (RR = 1.63, 95% CI = 0.75–3.52; RR = 1.36, 95% CI = 0.88–4.78) (Table 4).

**Fig. 1:**
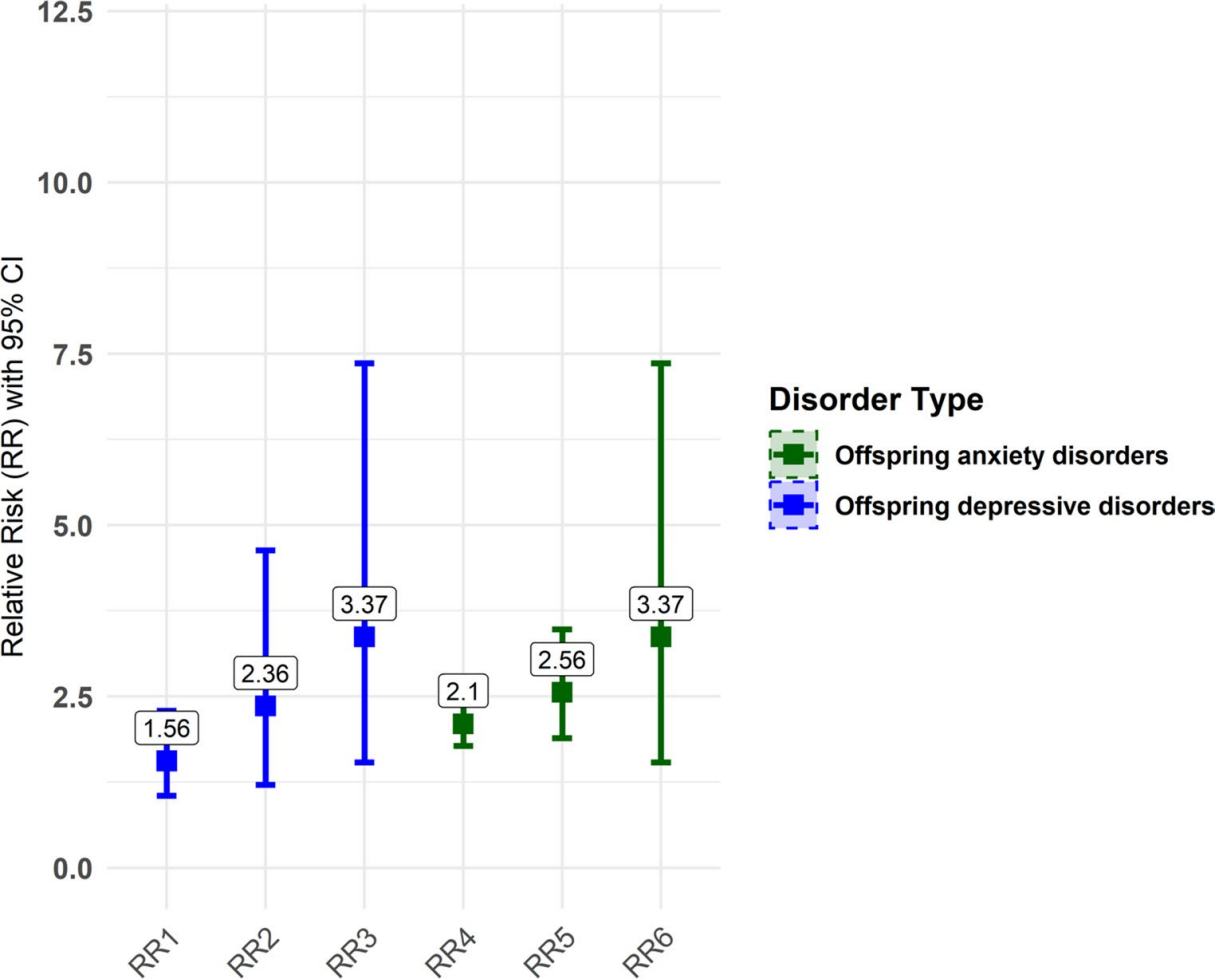
Maternal mental health comorbidity associated with higher risks of anxiety and depression in offspring. RR1: The risk of depressive disorders in the offspring of mothers with perinatal depressive disorders. RR2: The risk of depressive disorders in the offspring of mothers with comorbid perinatal depressive and anxiety disorders. RR3: The risk of comorbid depressive and anxiety disorder in the offspring of mothers with comorbid perinatal depressive and anxiety disorders. RR4: The risk of anxiety disorders in the offspring of mothers with perinatal depressive disorders. RR5: The risk of anxiety disorders in the offspring of mothers with comorbid perinatal depressive and anxiety disorders. RR6: The risk of comorbid anxiety and depressive disorder in the offspring of mothers with comorbid perinatal depressive and anxiety disorders

In this study, we observed an increasing relationship between maternal perinatal depression and anxiety and offspring depression and anxiety risks, with maternal mental health comorbidity associated with higher risks in offspring. Offspring of mothers with perinatal depressive disorder have a 1.56-fold higher risk of developing depressive disorder (RR = 1.56, 95% CI = 1.05–2.29) and a 2.10-fold higher risk of developing anxiety disorder (RR = 2.10, 95% CI = 1.78–2.48). When maternal depressive disorder co-occurs with anxiety, the risk increases to 2.36-fold for depressive disorder (RR = 2.36, 95% CI = 1.21–4.63) and 2.56-fold for anxiety disorder (RR = 2.56, 95% CI = 1.89–3.48). If both depression and anxiety are comorbid in the mother, the offspring’s risk of developing comorbid depression and anxiety disorders rises further to 3.37-fold (RR = 3.37, 95% CI = 1.54–7.36) (Fig. 1).

## Discussion

This study assessed the risk of depressive and anxiety disorders in the offspring of mothers with perinatal depressive disorders. The findings showed that children of mothers with perinatal depressive disorders were 1.56 to 3.37 times more likely to develop depressive and anxiety disorders, with stronger associations observed when maternal depressive and anxiety disorders co-occurred. These associations persisted after adjusting for potential confounders.

Our findings align with previous epidemiological research [24, 27, 31, 36], which has reported associations between maternal antenatal and postnatal depression and an increased risk of anxiety disorders in offspring. For example, cohort studies by Barker et al. [24] in the United Kingdom and Halligan et al. [27] in the United States reported that offspring of mothers with postnatal depression had more than a threefold higher likelihood of developing anxiety disorders. Similarly, a UK-based cohort study reported a 75% greater risk of anxiety disorders among offspring of mothers with antenatal depressive symptoms [25]. Furthermore, our study demonstrated that maternal perinatal depressive disorders were associated with an increased risk of specific anxiety disorders in offspring, including generalised anxiety disorder (GAD), consistent with findings from Lieb et al. [37], who reported a 2.30-fold higher risk of GAD in the offspring of mothers with depression. However, some studies have suggested no association between maternal postnatal depression and an increased risk of anxiety disorders in offspring [38, 39]. For example, research conducted by Bauer et al. in India [38] and a cohort study in the USA [39] found no evidence supporting this association. Notably, these studies used non-diagnostic screening tools such as the Center for Epidemiological Studies Depression (CES-D) and the Edinburgh Postnatal Depression Scale (EPDS) to assess maternal depression, which may have resulted in less accurate identification of depressive disorders. Additionally, smaller sample sizes in these studies could have limited their statistical power to detect a potential association.

Regarding depressive disorders, our study found that offspring of mothers with antenatal depressive disorders had a greater likelihood of being diagnosed with depressive disorders. However, maternal postnatal depression was not associated with an increased risk of offspring depression, and the significant association between antenatal depression and offspring depression diminished following covariate matching. This aligns with findings from other studies reporting no significant association between maternal antenatal or postnatal depression and offspring depression [27, 36, 40]. For instance, a UK-based cohort study did not observe an association between antenatal or postnatal depression and an increased risk of depressive symptoms in offspring [40]. Similarly, a cohort study by Halligan et al. in the USA reported no elevated risk of depressive disorders in offspring of mothers with postnatal depression [27].

Conversely, several studies have found a significant association between maternal antenatal and postnatal depression and the risk of offspring depression [24, 26–29, 41, 42]. For instance, cohort studies in the United Kingdom reported that offspring of mothers with antenatal or postnatal depression were more than three times as likely to develop depression [24, 42]. Discrepancies in findings may be attributed to differences in the timing of offspring depression assessments. Most previous studies measured depression in offspring at ages 12–18, a period when diagnosis is more common. In contrast, our study included offspring up to 15 years of age, potentially leading to fewer cases being captured, particularly among those who developed symptoms later in adolescence. This variation in outcome measurement timing likely explains the weaker associations observed in our study.

The mechanism through which maternal perinatal depression contributes to offspring depression and anxiety remains unclear, but several potential pathways have been proposed. Genetic predisposition may heighten offspring vulnerability to mental health disorders [6, 8, 9, 43]. Antenatal depression has been associated with elevated maternal cortisol levels [44–46], which can cross the placenta and influence fetal brain development [47], potentially altering stress response systems and increasing susceptibility to anxiety and depression [44]. From an environmental perspective, maternal depression, particularly during early childhood, may disrupt mother-child interactions, hinder emotional development, and elevate the risk of mental health disorders in offspring [48, 49]. Additionally, maternal depression is often accompanied by family stress, economic hardship, and social isolation, which may further compound mental health risks in offspring [50]. Epigenetic mechanisms may also play a role, as early exposure to maternal depression could trigger biological changes that shape long-term mental health outcomes in offspring.

Existing research has shown that maternal comorbid depression and anxiety heighten the risk of depressive and anxiety symptoms in offspring [25, 30]. An Australian birth cohort study reported that children of mothers with comorbid anxiety and depressive symptoms had a 63% greater likelihood of developing depressive symptoms by late adolescence [30]. Consistent with this, our study demonstrated that maternal comorbid perinatal depressive and anxiety disorders were more strongly associated with depressive, anxiety, and comorbid depressive and anxiety disorders in offspring, underscoring that the combined presence of maternal depression and anxiety has a more pronounced impact on offspring mental health than either condition alone. These associations remained consistent after covariate matching, possibly due to neurobiological mechanisms. Research on individuals with anxious depression has reported that hypothalamic-pituitary-adrenal (HPA) axis dysregulation is more severe in those with both anxiety and depression than in those with depression alone [51]. This heightened dysregulation results in more severe symptomatology, which may contribute to an increased risk of depression and anxiety in offspring [51]. Another potential mechanism underlying this association is the compounded effects of these conditions, which may lead to complex disruptions in the prenatal and postnatal environment, including heightened maternal stress, poorer overall health, and suboptimal caregiving, all of which may increase the risk of mental health disorders [52–54]. Additionally, the interplay of genetic predisposition and environmental stressors in mothers with comorbid anxiety and depressive symptoms may further contribute to the elevated risk in offspring [55]. It is also possible that maternal engagement with mental health services, which may be more common among those with comorbid conditions, contributed to the stronger associations observed, as these mothers might be more likely to seek care for both themselves and their children, thereby increasing the likelihood of diagnosis [56].

This study has several strengths. First, using ICD-10 diagnostic criteria for both exposure and outcome variables ensured clinical validity and consistency, minimising misclassification and enhancing the reliability of the findings. Second, the study provided a comprehensive evaluation of the combined effects of maternal perinatal depression and anxiety on offspring depressive and anxiety disorders, addressing a gap in previous research, which has primarily examined these conditions in isolation rather than their joint impact. Third, the large sample size improved statistical power, allowing for more precise effect estimates and strengthening the robustness of the findings. This also enhanced the generalisability of the results, making them more applicable to broader populations. Additionally, the study accounted for an extensive range of potential confounders, including socioeconomic factors, maternal physical health conditions, and other psychiatric comorbidities, which could otherwise distort associations. Covariate matching was applied to minimise bias and enhance comparability between exposure groups, reducing residual confounding and ensuring that observed associations were more likely to reflect true relationships rather than underlying differences between groups.

However, some limitations should be acknowledged. Residual confounding remains possible despite adjusting for multiple covariates, as not all relevant factors could be accounted for. Notably, data on paternal depression were unavailable, although evidence suggests its association with offspring depression and anxiety [57, 58]. Additionally, the study did not include information on maternal mental health prior to conception or beyond the perinatal period, limiting insight into its influence during other critical developmental stages. We also adjusted for maternal bipolar disorder and schizophrenia, given their independent associations with offspring mental health; however, this may have introduced over-adjustment bias, potentially attenuating the observed associations. The follow-up period of approximately 15 years allowed for tracking outcomes into adolescence; however, it may have been insufficient to capture the onset of depression and anxiety symptoms that emerge in later adolescence or early adulthood. This may have particularly affected the detection of depressive disorders, which tend to have a later onset than anxiety disorders, potentially influencing the observed patterns. Furthermore, our dataset does not include information on primary care provider visits or treatments, which may have resulted in the under-ascertainment of less severe maternal or offspring mental health conditions managed outside of hospital settings.

## Conclusion

Offspring of mothers with perinatal depressive disorders had an increased likelihood of developing depressive and anxiety disorders, with stronger associations observed when maternal depressive and anxiety disorders co-occurred. These associations persisted after adjusting for potential confounders and remained evident in PSM analyses. The findings highlight the potential long-term mental health risks for offspring and underscore the importance of early identification and targeted intervention for maternal perinatal depressive and anxiety disorders. Addressing these conditions during the perinatal period may help mitigate adverse psychiatric outcomes in offspring.

## Electronic supplementary material

Below is the link to the electronic supplementary material.

Supplementary Material 1

## Data Availability

No datasets were generated or analysed during the current study.
