# Supplementary material for: Anxiety and depressive disorders in the offspring of mothers with perinatal depressive disorders
Source: Eur Child Adolesc Psychiatry. 2025 Jul 25;34(12):4117–29. doi: 10.1007/s00787-025-02803-9 (PMC12743092; doi:10.1007/s00787-025-02803-9)
Supplement: Supplementary file 1 — Supplementary Material 1 [file 787_2025_2803_MOESM1_ESM.docx]

**Contents**

[Figure S1: The distribution of propensity scores in the treated and control groups (perinatal depressive disorders) 2](#_Toc189680584)

[Figure S2**:** The standardised mean differences for each covariate pre- and post-matching (perinatal depressive disorders) 3](#_Toc189680585)

[Figure S3 The distribution of propensity scores in the treated and control groups (antenatal depressive disorders) 4](#_Toc189680586)

[Figure S4: The standardised mean differences for each covariate pre- and post-matching (antenatal depressive disorders) 5](#_Toc189680587)

[Figure S5: The distribution of propensity scores in the treated and control groups (postnatal depressive disorders) 6](#_Toc189680588)

[Figure S6: The standardised mean differences for each covariate pre- and post-matching (postnatal depressive disorders) 7](#_Toc189680589)

[Figure S7: The distribution of propensity in the treated and control groups (Comorbid perinatal depressive and anxiety disorder) 8](#_Toc189680590)

[Figure S8: The standardised mean differences for each covariate pre- and post-matching (Comorbid perinatal depressive and anxiety disorder) 9](#_Toc189680591)


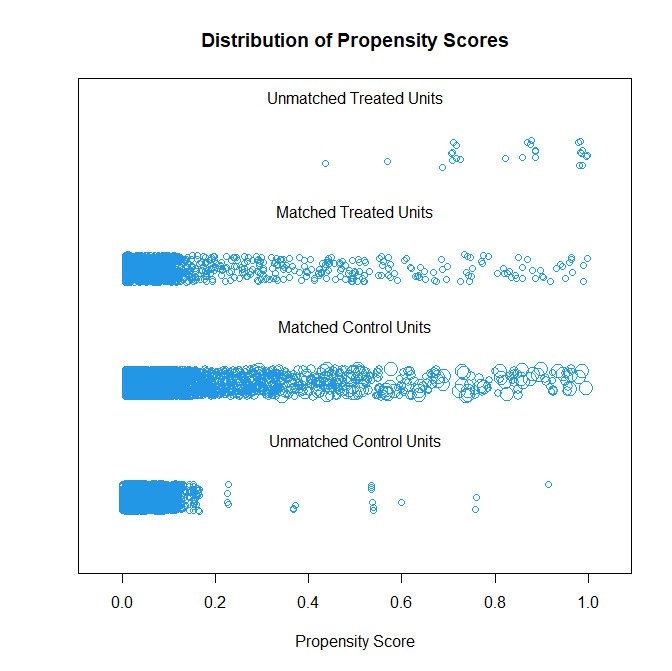


Figure S1: The distribution of propensity scores in the treated and control groups (perinatal depressive disorders)

Figure S2**:** The standardised mean differences for each covariate pre- and post-matching (perinatal depressive disorders)


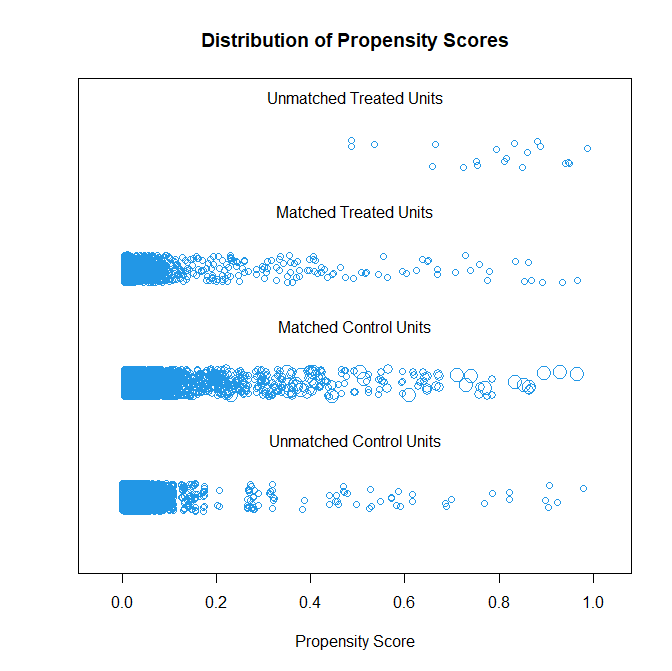


Figure S3 The distribution of propensity scores in the treated and control groups (antenatal depressive disorders)

Figure S4: The standardised mean differences for each covariate pre- and post-matching (antenatal depressive disorders)


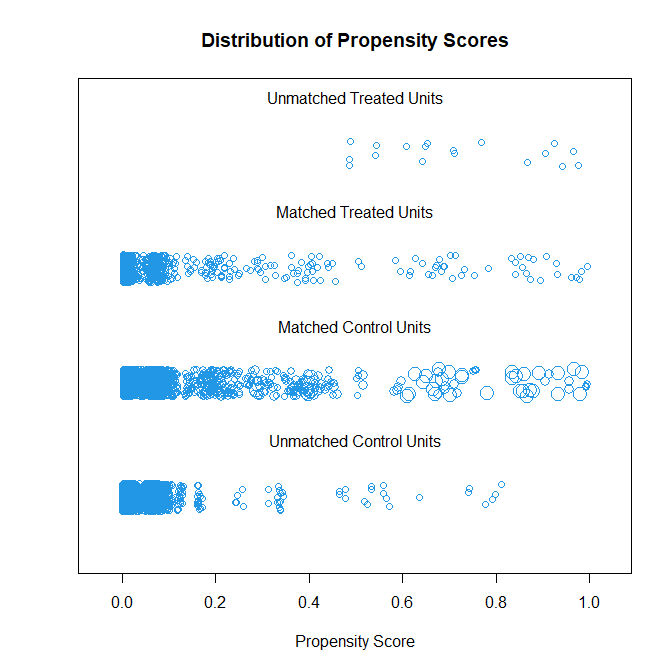


Figure S5: The distribution of propensity scores in the treated and control groups (postnatal depressive disorders)


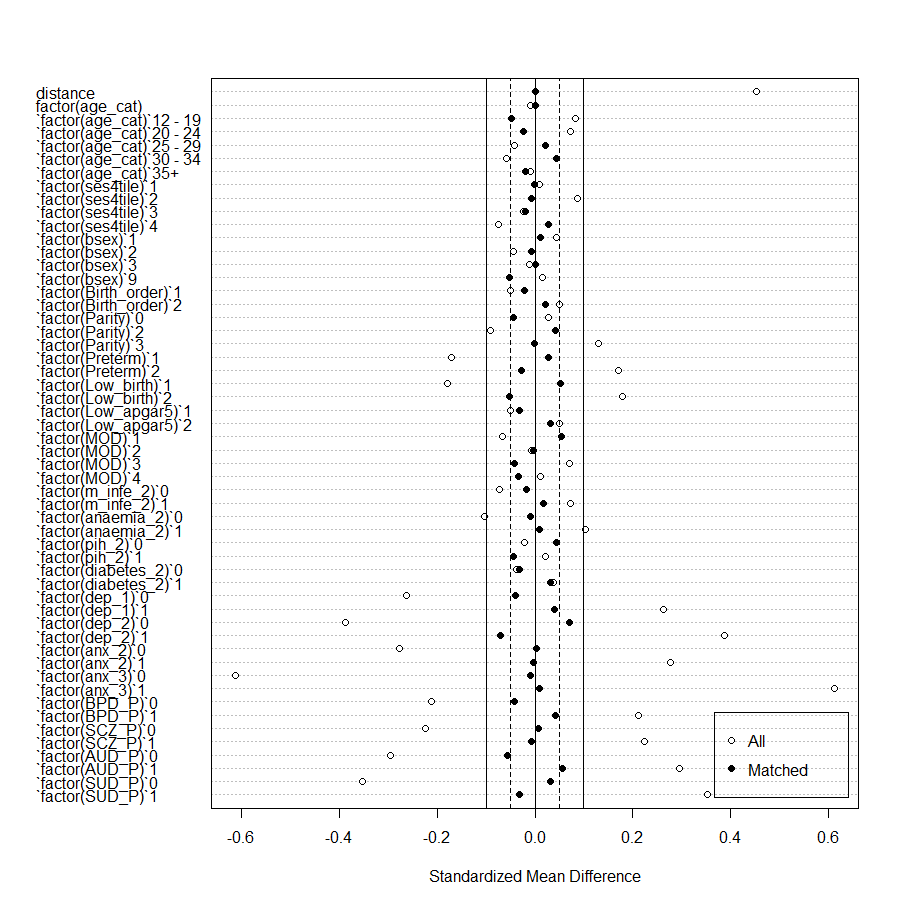


Figure S6: The standardised mean differences for each covariate pre- and post-matching (postnatal depressive disorders)


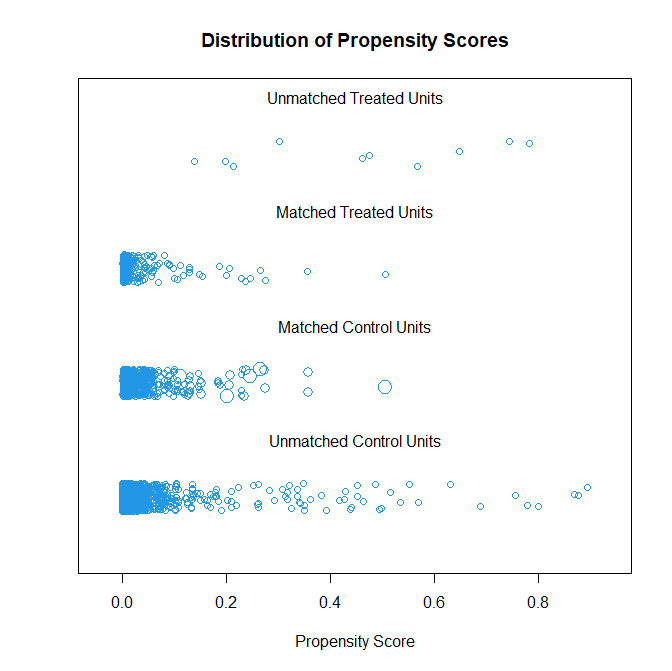


Figure S7: The distribution of propensity in the treated and control groups (Comorbid perinatal depressive and anxiety disorder)

Figure S8: The standardised mean differences for each covariate pre- and post-matching (Comorbid perinatal depressive and anxiety disorder)
